# Characterization of *Salmonella* spp. Isolates from European Hedgehogs (*Erinaceus europaeus*) in Italy: Serotypes and Antimicrobial Susceptibility Profiles

**DOI:** 10.3390/antibiotics15010046

**Published:** 2026-01-03

**Authors:** Sara Barbarulo, Elisa Rampacci, Sara Primavilla, Valentina Stefanetti, Fabrizio Passamonti

**Affiliations:** 1 Department of Veterinary Medicine, University of Perugia, Via San Costanzo 4, 06126 Perugia, Italy; sara.barbarulo@gmail.com (S.B.); elisa.rampacci@unipg.it (E.R.); fabrizio.passamonti@unipg.it (F.P.); 2 Istituto Zooprofilattico Sperimentale dell’Umbria e delle Marche “Togo Rosati”, Via G. Salvemini 1, 06126 Perugia, Italy; s.primavilla@izsum.it; 3 Wildlife Veterinary Research Center SELVA-VET, 06126 Perugia, Italy

**Keywords:** European hedgehogs, *Salmonella*, One Health, serotypes, antimicrobial resistance

## Abstract

Background: Wildlife is increasingly recognized as an important component in the epidemiology of zoonotic pathogens. *Salmonella* spp., a leading cause of foodborne disease worldwide, can circulate across human, domestic animal, and environmental interfaces. European hedgehogs (*Erinaceus europaeus*), a synanthropic species frequently inhabiting urban and peri-urban areas, may act as reservoirs or sentinels for *Salmonella*. Objectives: The aim of this study was to investigate the prevalence, serotype distribution, and antimicrobial susceptibility profiles of *Salmonella* spp. isolated from European hedgehogs admitted to wildlife rehabilitation centers in Italy. Methods: Fecal samples were collected from 100 European hedgehogs housed in five wildlife rehabilitation centers located in four Italian regions. *Salmonella* spp. were isolated using standard bacteriological methods, serotyped according to the Kaufmann–White–Le Minor scheme, and tested for antimicrobial susceptibility by broth microdilution for ampicillin, enrofloxacin, and sulfamethoxazole-trimethoprim. Minimum inhibitory concentrations (MICs) were interpreted following CLSI guidelines. Results: *Salmonella* spp. was isolated from 30% of the animals sampled. Four serovars were identified, with *S.* Enteritidis (50%) and *S.* Typhimurium (36.7%) being the most prevalent, followed by *S.* Agona (10%) and *S.* Chester (3.3%). Antimicrobial susceptibility testing revealed a high level of susceptibility, with 90% of isolates sensitive to all tested antibiotics. One *S. enteritidis* strain showed resistance to enrofloxacin and sulfamethoxazole–trimethoprim, while two isolates exhibited intermediate susceptibility to enrofloxacin. Conclusions: The detection of *Salmonella* serovars commonly associated with human infections in European hedgehogs highlights the potential role of this species in the ecology of zoonotic *Salmonella*. Although antimicrobial resistance levels were low, the presence of resistant and intermediate strains underscores the importance of continued surveillance. Despite some limitations related to the study design and sample representativeness, these results support the need for further large-scale investigations, reinforcing the need for integrated One Health surveillance strategies.

## 1. Introduction

In recent decades, urban expansion and the alteration of natural habitats have intensified interactions between humans and wildlife, thereby promoting the spread of zoonotic diseases [1,2]. Among these, salmonellosis is the second most prevalent zoonosis in the European Union, with over 91,000 cases reported annually, and it is the leading cause of foodborne outbreaks, according to the European Food Safety Authority (EFSA) and European Centre for Disease Prevention and Control (ECDC) [3]. Globally, it causes over 90 million cases and approximately 150,000 deaths each year [4]. *S. enterica* subsp. *enterica* serovar Typhimurium (*S*. Typhimurium) and *S.* Enteritidis are responsible for non-typhoidal forms with predominantly gastrointestinal manifestations. Other serovars relevant in human medicine are *S.* Infantis, *S.* Newport and *S.* Derby [3]. *Salmonella* spreads mainly via the fecal-oral route, through contaminated food, water, or contact with infected animals. *Salmonella* spp. is recognized as capable of infecting a wide range of host species, including food-producing and companion animals, as well as wildlife [5]. From a One Health perspective, the circulation of *Salmonella* across different ecological compartments represents a relevant public health concern, as wildlife may act as reservoirs, sentinels, or spillover hosts at the human–animal–environment interface [6]. In several European countries, *Salmonella* surveillance and control programs are primarily focused on food-producing animals due to their direct role in food safety and human exposure [3]. In contrast, the monitoring of *Salmonella* in wild animals is not routinely included in national surveillance systems and is generally limited to sporadic investigations conducted for research purposes [7,8,9]. Although several studies have addressed the presence and epidemiology of *Salmonella* in wild birds [10,11], comparatively limited information is available regarding its occurrence, ecological role, and potential public health implications in wild mammal populations, including the European hedgehog (*Erinaceus europaeus*) [12,13]. A quintessential synanthropic species, the hedgehog has progressively adapted to urban and suburban environments, where it shares space with humans and domestic animals [14]. Moreover, hedgehogs are often infested with ticks and ectoparasites [15,16] and may contribute to maintaining ticks in suburban areas, especially in winter [17]. Hedgehogs’ interaction with several animals and their high population density make them promising candidates for spreading zoonotic pathogens, including *Salmonella* spp., whose non-foodborne routes of transmission have been documented in recent phylogenetic studies [3]. These studies revealed genetic similarities between strains isolated from hedgehogs and humans, suggesting potential interspecies transmission, particularly in rural areas of the United Kingdom [18]. Importantly, analyzing *Salmonella* isolates from wildlife for the presence of antimicrobial resistance (AMR) yields information about the extent of the environmental pollution [19]. Several free-ranging species have been investigated to rule out their role in antibiotic resistance, and the most represented group of animals in scientific literature is wild birds [20]. Uelze et al. analyzed, through an in silico approach, the distribution of *Salmonella* serovars across the different wildlife species, such as wild boar, red foxes, deer, hedgehogs, raccoons, badgers, wildcats, and wolves. The most found serovars were *S.* Choleraesuis, *S.* Enteritidis and *S.* Typhimurium with a low AMR burden [13].

The actual role of the hedgehog in the dissemination of *Salmonella* spp. remains poorly understood, especially in continental Europe. This knowledge gap highlights the need for integrated surveillance strategies that include humans, domestic animals, and wildlife in order to better understand transmission dynamics and antimicrobial resistance dissemination within a One Health context [21,22,23]. This study aims to investigate the prevalence of *Salmonella* spp. and its antimicrobial susceptibility profiles in Italian European hedgehogs.

## 2. Results

This study examined the presence and distribution of *Salmonella* spp. serotypes in European hedgehogs admitted to wildlife rehabilitation centers in Lazio, Umbria, Tuscany, and Piedmont. Between 2023 and 2024, *Salmonella* spp. was isolated from 30 out of 100 animals (30% prevalence). Among the positive samples, four different *Salmonella* serovars were identified, as reported in Table 1.

*S.* Enteritidis was the most frequently isolated serotype, accounting for 50% of the cases, followed by *S.* Typhimurium (36.7%), *S.* Agona (10%), and *S.* Chester (3.3%).

**Figure 1:**
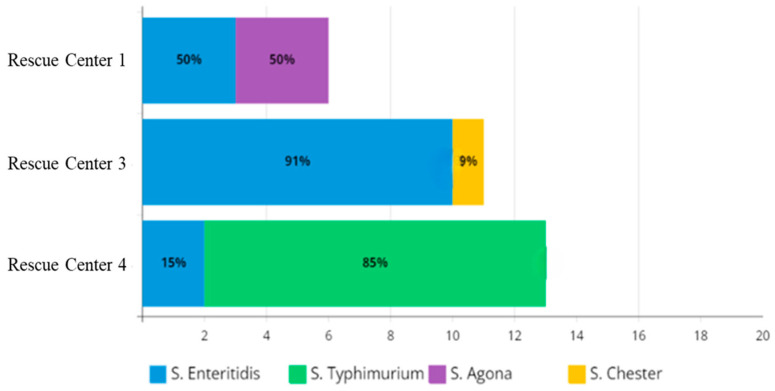
Distribution of serovars of *Salmonella enterica* subsp. *enterica* across different rescue centers.

Figure 1 shows the prevalence of serovars for Wildlife Rescue Centers 1, 3 and 4. No *Salmonella* species were isolated in Rescue Center 2 and the University Veterinary Teaching Hospital of Perugia (UVTH).

Broth microdilution testing revealed high antibiotic susceptibility: 90% of *Salmonella* isolates were sensitive to all antibiotics tested. One *S. enteritidis* strain, isolated from a hedgehog housed at Rescue Center 1, was found to be resistant to both enrofloxacin and sulfatrimethoprim. MIC testing by broth microdilution revealed values of 4 µg/mL for enrofloxacin and 10 µg/mL for sulfatrimethoprim. Two isolates, corresponding to *S. enteritidis* and *S. chester*, showed intermediate susceptibility to enrofloxacin. The *S. enteritidis* isolate, obtained from a sample provided by Rescue Center 1, exhibited a MIC value of 2 µg/mL, while the *S. chester* isolate, derived from a specimen received from Rescue Center 3, showed a MIC value of 1 µg/mL. Detailed results of the broth microdilution test, including MIC values for each isolate, are reported in Table 2. No statistically significant differences were identified among the susceptibility patterns and the rescue centers.

## 3. Discussion

Salmonellosis is one of the most widespread zoonoses globally. The analysis of collected data helps to outline the epidemiology of *Salmonella* spp. in the European hedgehog population, highlighting both the pathogenic potential of this bacterium and its ability to develop antibiotic resistance.

The prevalence observed in the present study aligns with previous findings in Emilia-Romagna, which reported a 39% prevalence from 2019 to 2022 [24], although it may still be underestimated.

Most Italian epidemiological studies focus on northern regions, while the south is largely neglected. However, the lack of data prevents a full understanding of the real epidemiological situation in these areas. Further investigations into the distribution of *Salmonella* serotypes circulating in urban wildlife would be advisable to fill this geographical and ecological gap.

Considering the overall prevalence of serotypes, *S.* Enteritidis and *S.* Typhimurium were the most isolated, accounting for 50% and 36.7% of cases, respectively.

The survey conducted between 2019 and 2022 on hedgehogs at the Wildlife Rescue Center 1 in Ferrara confirms *S. enteritidis* as the predominant serotype [24]. The isolation of *S.* Enteritidis and *S.* Typhimurium in this study is particularly relevant, since these serotypes are not host-specific and can infect a wide range of species, facilitating environmental persistence [25]. Uelze et al. investigated through Whole Genome Sequencing (WGS) *Salmonella enterica* subsp. *enterica* in several wildlife species, including badgers, deer, hedgehogs, pigeons, raccoons, wild birds, wild boar, wild cats, and foxes. They found *S.* Choleraesuis, *S.* Enteritidis and *S.* Typhimurium as the most common serovars. Interestingly, within these serovars, different sequence types were found to be associated with different animal species. All hedgehog isolates belonged to *S.* Enteritidis ST183 [13].

Scientific literature reports numerous cases of human illness linked to these serotypes, often following consumption of contaminated food or contact with pet animals carrying bacteria. Likewise, African pygmy hedgehogs have also been recognized as vectors [26]. Their growing popularity as pets has raised health and safety concerns; in 2020, they were identified as the primary source of *S.* Typhimurium outbreaks in the United States and Canada [27].

Only a strain of *S.* Chester was reported in our study. Major foodborne outbreaks caused by *S.* Chester in humans have been documented in Australia [28], Japan [29], and Canada [30]. Data on the prevalence of *S.* Chester in animals intended for human consumption indicated the presence of this bacterium in camel meat [31].

However, particular interest was raised by an analysis of *Salmonella* prevalence in the feces of wild hedgehogs, which in Burkina Faso are also a food source for the local population. Although the prevalence of *Salmonella* in hedgehogs was high (96%), the *S.* Chester serotype was not reported in this species [32].

In hedgehogs from Rescue Center 1 in Rome, three *S.* Agona were identified.

*S.* Agona serotype, rarely isolated in humans, is often associated with contamination of animal feed and food products [33].

The role of wildlife in *Salmonella* transmission is less clear, mainly due to the difficulty in establishing direct connections between human infections and wild animal contact. Nevertheless, animals in wildlife rescue centers require constant care, increasing the likelihood of human–animal interaction. *Salmonella* can persist in the environment by producing biofilms [34]. Biofilms allow the pathogen to survive on inanimate surfaces (fomites), such as cages, bedding, food containers, and medical equipment, promoting indirect transmission. Therefore, strict hygiene protocols are essential in managing captive wild animals to prevent outbreaks. This includes regular cleaning, frequent bedding changes, and the use of personal protective equipment, which should be replaced among animals to avoid cross-contamination. Proper implementation of these measures reduces the animals’ susceptibility to infection and the need for medical treatment. Minimizing antibiotic use is crucial to preserve their effectiveness.

The global concern over antimicrobial resistance is growing. In wildlife centers, the repeated use of common antibiotics—often due to a limited range of approved drugs—encourages the selection of resistant strains [35]. Nonetheless, this study shows high levels of susceptibility, possibly linked to inherent characteristics of *Salmonella* phage types found in European hedgehogs [27]. Our results are in line with previous studies: Lawson et al. found 46 hedgehog isolates investigated susceptible to antibiotics [18], and no resistance genes were observed in isolates obtained from hedgehogs by Uelze and collaborators [13]. Still, strains with intermediate resistance must be monitored, as they could evolve toward full resistance. The confined conditions in shelters facilitate high bacterial loads and create ideal conditions for genetic exchange among microorganisms. These findings reinforce the need for ongoing surveillance and cautious antibiotic use in wildlife rehabilitation settings.

Further sampling and genomic sequencing are essential to define the circulating genotypes of *Salmonella* spp. and to determine any biological similarities between strains found in hedgehogs. Comparative studies involving African pygmy hedgehogs would also be highly informative due to their evolutionary proximity to *Erinaceus* species and possible pathogen overlap. Exploring these similarities could enhance understanding of cross-species transmission and help identify potential emerging zoonoses. Advancing this research is crucial for improving our grasp of host–pathogen interactions and for designing effective disease prevention strategies.

## 4. Conclusions

This study highlights the presence of *Salmonella* spp. in hedgehogs temporarily housed in wildlife rehabilitation centers, confirming their potential role as reservoirs of zoonotic pathogens. Although we found low antimicrobial-resistant strains, our findings provide valuable baseline data and underscore the importance of integrating wildlife into One Health surveillance programs. Further large-scale and longitudinal studies are warranted to better assess the epidemiological significance of hedgehogs in the maintenance and transmission of *Salmonella* and antimicrobial resistance.

## 5. Materials and Methods

### 5.1. Sample Collection and Bacterial Isolation

**Figure 2:**
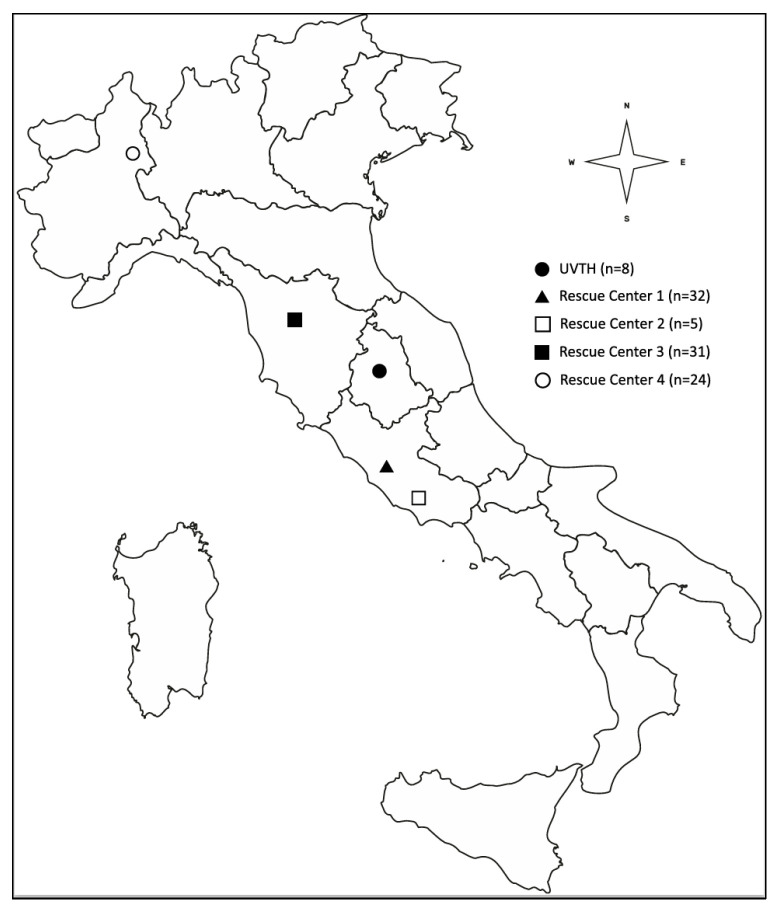
Geographical origin of the animals and number of hedgehogs sampled in each sampling center.

A total of 100 fecal samples were collected over a four-month period, from December 2023 to March 2024, from European hedgehogs housed in five wildlife rehabilitation centers located in four Italian regions. The animals examined were hedgehogs originating from their natural environment and only temporarily admitted to wildlife rehabilitation centers. Their stay was limited in duration, ranging from a few days to several months, depending on the individual case. Animals originated from the University Veterinary Teaching Hospital of Perugia (UVTH), Umbria (n = 8); Rescue Center 1 in Rome, Lazio (n = 32); Rescue Center 2 in Latina, Lazio (n = 5); Rescue Center 3 in Montespertoli (FI), Tuscany (n = 31); and Rescue Center 4 in Novara, Piedmont (n = 24), located as shown in Figure 2.

Most individuals were housed in separate cages within dedicated indoor facilities, except for those from Rescue Center 1, which were moved to outdoor shared enclosures prior to release. At this center, higher levels of interaction between hedgehogs and wild birds were also observed. To reduce potential bias, only hedgehogs that had not received antibiotic treatment in the two weeks prior to sampling were included, except for those from UVTH, which had been treated with enrofloxacin for trauma-related conditions.

### 5.2. Bacteriological Examination

Fresh fecal samples were collected using sterile dry cotton swabs, stored under refrigeration for a maximum of 48 h, and submitted to the Microbiology Laboratory of the Department of Veterinary Medicine, University of Perugia, for bacteriological examination.

The protocol adopted for the isolation of *Salmonella* spp. was performed according to the standard procedures [36]. Initially, 10 mL of Buffered Peptone Water (BPW) Liofilchem^®^ (Roseto degli Abruzzi, Teramo, Italy) was added to the fecal swabs, which were then incubated at 37 °C ± 1 °C for a period ranging from 16 to 20 h. After this pre-enrichment phase, 0.1 mL of the culture was transferred into 10 mL of Rappaport Vassiliadis Broth Liofilchem^®^ and incubated at 42 °C ± 1 °C for 18 to 24 h. For the isolation step, 0.01 mL of the broth culture was taken using a bacteriological loop and streaked onto solid selective-differential agar media, specifically XLD agar Liofilchem^®^ and Chromogenic Salmonella agar (CSA) Liofilchem^®^, to obtain isolated colonies. The plates were then incubated at 37 °C ± 1 °C for 18 to 24 h under aerobic conditions.

*Salmonella* spp. isolates were then serotyped by direct slide agglutination using specific antisera (Statens Serum Institute, Copenhagen, Denmark) to detect somatic (O) and flagellar (H) antigens. The analysis was performed according to the Kaufmann-White-Le Minor scheme, as described in ISO/TR 6579-3:2014, at the Istituto Zooprofilattico Sperimentale dell’Umbria e delle Marche “Togo Rosati”. When necessary, the Sven Gard method was used to perform phase inversion to detect the inapparent H antigen phase of biphasic *Salmonella* [36].

### 5.3. Antimicrobial Susceptibility Testing

*Salmonella* strains were subjected to antimicrobial susceptibility testing (AST) using the broth microdilution method. The antibiotics tested were selected according to CLSI guidelines [37]. Specifically, ampicillin was used to represent the β-lactam class, enrofloxacin for the quinolones, and sulfamethoxazole-trimethoprim for the sulfonamides.

Briefly, two-fold drug dilutions were performed in Cation-Adjusted Mueller Hinton Broth (CAMHB) to test the following concentration ranges: 256–2 μg/mL for ampicillin, 32–0.25 μg/mL for enrofloxacin, and 640–5 μg/mL for sulfamethoxazole-trimethoprim. Bacterial inoculum was diluted in CAMHB starting from a suspension at the spectrophotometric 0.5 McFarland standard to obtain a final concentration of 5 × 10^5^ CFU/mL (5 × 10^4^ CFU/well). Positive (bacterial inoculum without drug) and negative control (sterile CAMHB) wells were tested in each plate, then incubated at 37 °C. The standard reading was established at 24 h, and all the experiments were performed in triplicate. A preliminary quality control test was performed before the experiments using the *S. enteritidis* reference strain ATCC 13076. The interpretation of MIC values (expressed in μg/mL) was based on the clinical breakpoints established by the CLSI for *Enterobacteriaceae* [37].

### 5.4. Statistical Analysis

Data were analyzed using commercially available statistical software (JASP, JASP Team 2022, version 0.16.1). Fisher’s exact test was applied to identify differences in antimicrobial susceptibility between the different rescue centers, as indicated. Statistical significance was set at a *p*-value of less than 0.05.

## Figures and Tables

**Table 1:** *Salmonella enterica* subsp. *enterica* serovar isolated from fecal samples of hedgehogs housed in three rescue centers in Italy.

N. Sample	Region	Serovars
1	Lazio	*S.* Agona
2	Lazio	*S.* Enteritidis
3	Lazio	*S.* Agona
4	Lazio	*S.* Agona
5	Lazio	*S.* Enteritidis
6	Lazio	*S.* Enteritidis
7	Tuscany	*S.* Chester
8	Tuscany	*S.* Enteritidis
9	Tuscany	*S.* Enteritidis
10	Tuscany	*S.* Enteritidis
11	Tuscany	*S.* Enteritidis
12	Tuscany	*S.* Enteritidis
13	Tuscany	*S.* Enteritidis
14	Tuscany	*S.* Enteritidis
15	Tuscany	*S.* Enteritidis
16	Tuscany	*S.* Enteritidis
17	Tuscany	*S.* Enteritidis
18	Piedmont	*S.* Typhimurium
19	Piedmont	*S.* Typhimurium
20	Piedmont	*S.* Typhimurium
21	Piedmont	*S.* Typhimurium
22	Piedmont	*S.* Enteritidis
23	Piedmont	*S.* Typhimurium
24	Piedmont	*S.* Typhimurium
25	Piedmont	*S.* Typhimurium
26	Piedmont	*S.* Typhimurium
27	Piedmont	*S.* Typhimurium
28	Piedmont	*S.* Typhimurium
29	Piedmont	*S.* Typhimurium
30	Piedmont	*S.* Enteritidis

**Table 2:** MIC values for each isolate according to CLSI guidelines.

N. Sample	Ampicillin	Enrofloxacin	Sulfatrimethoprim
1	2 µg/mL (S)	≤0.25 µg/mL (S)	≤5 µg/mL (S)
2	8 µg/mL (S)	4 µg/mL (R)	10 µg/mL (R)
3	≤2 µg/mL (S)	≤0.25 µg/mL (S)	≤5 µg/mL (S)
4	≤2 µg/mL (S)	≤0.25 µg/mL (S)	≤5 µg/mL (S)
5	4 µg/mL (S)	≤0.25 µg/mL (S)	≤5 µg/mL (S)
6	4 µg/mL (S)	2 µg/mL (I)	≤5 µg/mL (S)
7	≤2 µg/mL (S)	1 µg/mL (I)	≤5 µg/mL (S)
8	≤2 µg/mL (S)	≤0.25 µg/mL (S)	≤5 µg/mL (S)
9	≤2 µg/mL (S)	≤0.25 µg/mL (S)	≤5 µg/mL (S)
10	≤2 µg/mL (S)	≤0.25 µg/mL (S)	≤5 µg/mL (S)
11	≤2 µg/mL (S)	≤0.25 µg/mL (S)	≤5 µg/mL (S)
12	≤2 µg/mL (S)	≤0.25 µg/mL (S)	≤5 µg/mL (S)
13	≤2 µg/mL (S)	≤0.25 µg/mL (S)	≤5 µg/mL (S)
14	≤2 µg/mL (S)	≤0.25 µg/mL (S)	≤5 µg/mL (S)
15	≤2 µg/mL (S)	≤0.25 µg/mL (S)	≤5 µg/mL (S)
16	≤2 µg/mL (S)	≤0.25 µg/mL (S)	≤5 µg/mL (S)
17	≤2 µg/mL (S)	≤0.25 µg/mL (S)	≤5 µg/mL (S)
18	≤2 µg/mL (S)	≤0.25 µg/mL (S)	≤5 µg/mL (S)
19	≤2 µg/mL (S)	≤0.25 µg/mL (S)	≤5 µg/mL (S)
20	≤2 µg/mL (S)	≤0.25 µg/mL (S)	≤5 µg/mL (S)
21	≤2 µg/mL (S)	≤0.25 µg/mL (S)	≤5 µg/mL (S)
22	≤2 µg/mL (S)	≤0.25 µg/mL (S)	≤5 µg/mL (S)
23	≤2 µg/mL (S)	≤0.25 µg/mL (S)	≤5 µg/mL (S)
24	≤2 µg/mL (S)	≤0.25 µg/mL (S)	≤5 µg/mL (S)
25	≤2 µg/mL (S)	≤0.25 µg/mL (S)	≤5 µg/mL (S)
26	≤2 µg/mL (S)	≤0.25 µg/mL (S)	≤5 µg/mL (S)
27	4 µg/mL (S)	≤0.25 µg/mL (S)	≤5 µg/mL (S)
28	≤2 µg/mL (S)	≤0.25 µg/mL (S)	≤5 µg/mL (S)
29	4 µg/mL (S)	≤0.25 µg/mL (S)	≤5 µg/mL (S)
30	≤2 µg/mL (S)	≤0.25 µg/mL (S)	≤5 µg/mL (S)

S: sensitive; I: intermediate; R: resistant.

## Data Availability

The original contributions presented in the study are included in the main article; further inquiries can be directed at the corresponding author.
